# Physiological Effects of Exogenously Applied Reflectants and Anti-Transpirants on Leaf Temperature and Fruit Sunburn in Citrus

**DOI:** 10.3390/plants8120549

**Published:** 2019-11-27

**Authors:** Julissa Rodriguez, Ambrose Anoruo, John Jifon, Catherine Simpson

**Affiliations:** 1 Department of Agriculture, Agribusiness, and Environmental Science, Texas A&M University Kingsville-Citrus Center, 312 N. International Blvd., Weslaco, TX 78599, USA; julissa.rodriguez@students.tamuk.edu; 2 Department of Agriculture, Agribusiness, and Environmental Sciences, Texas A&M University-Kingsville, 700 University Boulevard, MSC 228, Kingsville, TX 78363, USA; ambrose.anoruo@tamuk.edu; 3 Department of Horticultural Sciences, Texas A&M AgriLife Research, 2415 E. Business 83, Weslaco, TX 78596, USA; jifon@tamu.edu

**Keywords:** sunburn, citrus, Lower Rio Grande Valley, reflectants, calcium, anti-transpirants

## Abstract

High temperatures and drought are common stresses limiting crop growth and productivity in subtropical regions where citrus are produced. In addition to impacts on physiological processes such as transpiration, photosynthesis, and respiration, excessive solar radiation can also reduce fruit productivity by inducing physiological disorders such as sunburn. This study evaluated the effects of radiation reflectants and anti-transpirants on leaf physiology, and fruit sunburn in grapefruit trees (*Citrus x paradisi* Macfs. cv. Rio Red) in south Texas during the 2016 and 2017 growing seasons. Two calcium-based reflectants, and a methene/pinolene-based anti-transpirant were foliar applied to fruit-bearing trees. Reflectants reduced fruit and leaf temperatures by 0.2 °C and 0.21 °C, respectively, while the anti-transpirant treatments increased fruit and leaf temperature by approximately 0.83 °C and 0.2 °C relative to the controls. Stomatal conductance decreased by 1.3% and 3.3%, respectively, in response to the reflectant treatments, while anti-transpirant treatments resulted in decreased stomatal conductance (8.3%) relative to the controls. More sunburned fruit were found in anti-transpirant treated trees in both years (6% and 8.2% for 2016 and 2017) and the reflectant treatments reduced sunburn incidence by 4.9% and 1.8% in those years. These observations indicate that reflectant applications could be a viable strategy to mitigate heat/radiation stress and sunburn in grapefruit.

## 1. Introduction

Sunburn is a physiological disorder in citrus and other fruit species caused by excess light and solar radiation [1]. Studies on reducing sunburn in citrus have not been extensive as most of the research to reduce the incidence of sunburn, canopy temperatures, and water stress have been conducted in apples, pomegranates, pears, and loquat [2,3,4]. It is well established that chemical reflectants and kaolin sprays can reduce sunburn in sensitive fruits [2,3]. In addition, anti-transpirants can be used to minimize water loss in times of drought or heat stress [2]. However, the physiological impacts and interactions of these compounds on citrus have not been studied thoroughly. Sunburned fruit is discolored and exhibits varying degrees of cell death [1,2]. Fruit sunburn is a result of the high fluctuation densities of solar radiation that affect the natural defense systems of plants, causing commercial losses of fruits and vegetables [1,2,3,4]. Sunburn is particularly problematic in arid and semi-arid regions that have high solar radiation. In regions that experience a high incidence of fruit sunburn due to these conditions, the likelihood of plant water stress due to heat and drought stress is also higher. While thin skinned fruit is considered to be more susceptible and fruit quality and marketability is more affected, other fruit crops can also suffer. Sunburn in citrus fruit has been a common physiological issue facing growers and causes financial losses each year [1]. Studies on sunburn and water stress management include the application of foliar sprays with inert reflective materials such as kaolinite clay [5,6,7], anti-transpirants, evaporative cooling systems [8,9], and protecting fruit with paper bags to reduce the appearance of sunscald [2,10]. Kaolin treatment has been used for sunburn reduction with some success on apples [11], grapefruit [12], and pomegranates [13]. Washington State produces over half of the apple crop in the United States, yet sunburn causes serious economic losses averaging over 10% annually [9]. In Australia, sunburn causes 40–50% of losses for sensitive apple cultivars [14] and in Taiwan, ‘Murcott’ tangerines and some early clementine mandarins are susceptible to sunburn, causing 13.6% of losses [10]. In most of these regions, few or no mitigation practices are currently in place to prevent fruit losses due to sunburn.

The development of sunburn has been attributed to the combination of visible light and high temperatures [15]. Rabinowitch et al. (1974) [15] reported that heat and visible light are essential for the development of common sunburn symptoms in tomatoes. Additionally, Rabinowitch et al. (1986) [16] also reported that air temperature thresholds exceeding 38 °C were critical for the development of sunburn injury in peppers and cucumbers. In apples, sunburn disrupts the structure and morphology of the fruit, alters pigment composition, and decreases photosynthesis, which results in decreased fruit quality. Similarly, in citrus fruits, sunburn alters photosynthetic systems and ruptures oil glands, leading to subsequent water loss and reductions in growth and yield [10,17]. The combination of high irradiance and high temperatures causes the formation of highly reactive and hazardous active oxygen species (AOS) in plant tissue [18,19]. The free radicals cause a loss of membrane integrity that leads to electrolyte leakage that affects the cell lipids and proteins, ultimately killing the cell [19,20]. In citrus, sunburn is enhanced by the deterioration of the oil glands in the flavedo, which injures underlying cells [21]. Different types of sunburn (sunburn necrosis, sunburn browning, and photo-oxidative sunburn) have been categorized and characterized in apples [9,22,23]. However, exposure to sun in citrus can result in discoloration of the fruit, ranging from light yellowing or bleaching to dark necrotic spots [1].

Fruit sunburn disorder has been reported to be more severe in stressed trees, particularly those that are water stressed and on trees lacking vigor [1,22,24,25]. Increased fruit flesh firmness, dry matter content, and reduced relative water content and titratable acidity (TA) have been reported in sunburned fruit [26]. These factors contribute to the decline in fruit quality, total financial impact of fruit sunburn, and losses by producers. Reducing plant water stress and using shade nets and particle films are some of the mitigation options currently used in commercial orchards [27]. Therefore, the objectives of these studies were to evaluate the environment in which sunburn develops in grapefruit and compare different treatments using reflectants (R) and anti-transpirants (AT) in order to determine any beneficial or detrimental effects on trees and fruit quality. 

## 2. Results

### 2.1. Environmental Conditions

**Figure 1:**
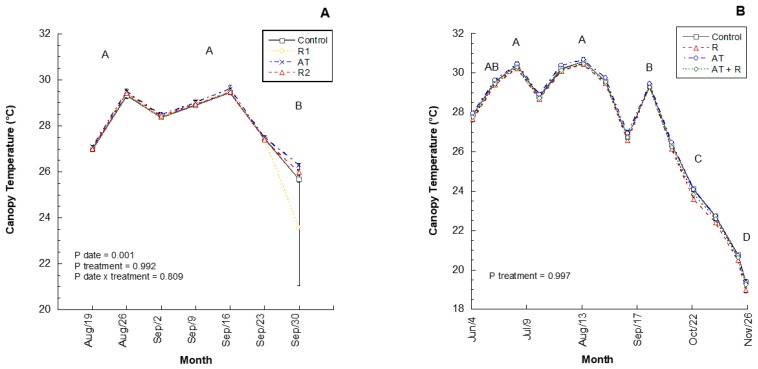
2016 and 2017 by weekly canopy temperatures (°C). (**A**) Average canopy temperature (°C) recorded bi-weekly from August to September 2016. 2016 Treatments: R1 = Reflectant at 9.35 L/ha; R2= Reflectant at 18.7 L/ha; AT = Anti-transpirant at 14 L/ha. (**B**) Average canopy temperature (°C) recorded by weekly from June to November 2017. 2017 Treatments: R = Reflectant at 9.35 L/ha; AT = Antitranspirant at 9.35 L/ha; AT + R = Anti-transpirant at 9.35 L/ha and Reflectant at 18.7 L/ha. Error bars represent ±1 standard error of the mean. Significant difference between months are shown as different uppercase letters.

In 2016, two rates of a calcium-based reflectant (Reflectant 1, R1; Reflectant 2, R2), an anti-transpirant (AT), and a water control were applied to trees. In this experimental year, there were no significant treatment effects on the average weekly canopy air temperatures (Figure 1A; *p* = 0.9916). However, canopy temperatures differed significantly between months (*p* = 0.0005), wherein August and September had the highest canopy temperatures and October had the lowest temperatures. There was no significant difference between treatments by month (*p* = 0.8099). In 2017, the products were used again, but in different combinations and rates: anti-transpirant (AT), reflectant (R), anti-transpirant and reflectant (AT + R), and a control. Similar to the 2016 results, in 2017, the averaged weekly data showed that treatment did not have a significant effect on temperature within the canopy (Figure 1B; *p* = 0.9974). However, canopy temperature showed a significant difference between months (*p* = 0.0001), where July and August had the highest canopy temperatures followed by June and September, October and November. There was no significant interaction between treatment and month.

**Figure 2:**
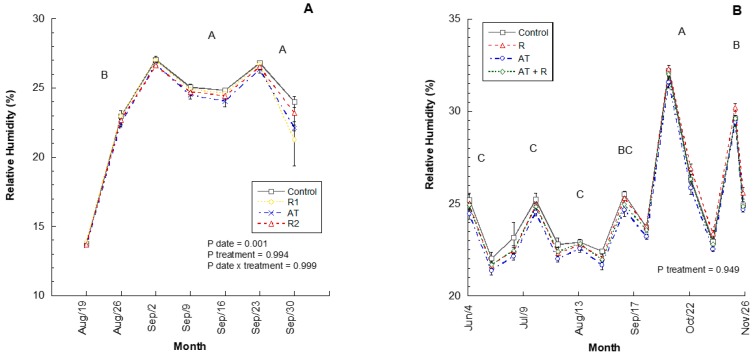
Average canopy relative humidity for the 2016 and 2017 experiments. (**A**) Average % relative humidity recorded from August to October 2016. 2016 Treatments: R1 = Reflectant at 9.35 L/ha; R2 = Reflectant at 18.7 L/ha; AT = Anti-transpirant at 14 L/ha. (**B**) Average % relative humidity recorded from June to November 2017. 2017 Treatments: R = Reflectant at 9.35 L/ha; AT = Anti-transpirant at 9.35 L/ha; AT + R = Anti-transpirant at 9.35 L/ha and Reflectant at 18.7 L/ha. Bars represent ±1 standard error of the mean. Uppercase letters show significant differences between months.

In 2016 and 2017, relative humidity within the canopy was not significantly different between treatments (Figure 2; *p* = 0.9935, 0.9494; respectively). However, relative humidity was statistically different each month for 2016 (*p* = 0.0004); where September and October had higher relative humidity when compared to August. In 2017, the relative humidity was statistically different for each month (*p* = 0.0001), where October and November showed a higher relative humidity, followed by September, and the months of June, July, and August had the lowest values. There was no interaction between treatment and month for 2016 or 2017 (Figure 2A,B).

### 2.2. Tree Measurements

#### 2.2.1. Stomatal Conductance

**Figure 3:**
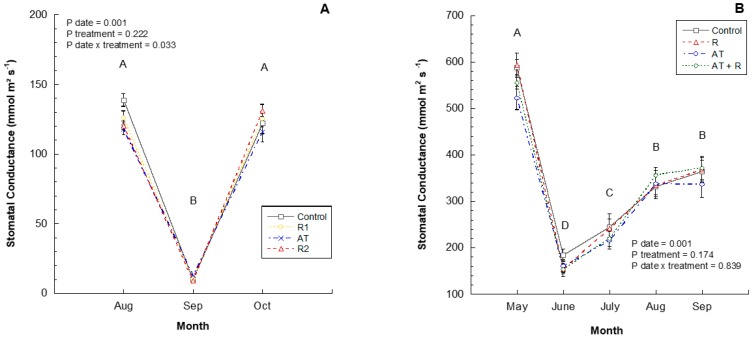
2016 and 2017 stomatal conductance (mmoL m^2^s^−1^). (**A**) Stomatal conductance (mmoL m^2^s^−1^) from August to October 2016. 2016 Treatments: R1 = Reflectant at 9.35 L/ha; R2 = Reflectant at 18.7 L/ha; AT = Anti-transpirant at 14 L/ha. (**B**) Stomatal conductance (mmoL m^2^s^−1^) from May to September 2017. 2017 Treatments: R = Reflectant at 9.35 L/ha; AT = Anti-transpirant at 9.35 L/ha; AT + R = Anti-transpirant at 9.35 L/ha and Reflectant at 18.7 L/ha. Bars represent ±1 standard error of the mean. Uppercase letters show significant differences between months.

In 2016, the treatments significantly affected stomatal conductance at each measurement date (Figure 3A). In August, the leaves of the control treatment trees had the highest stomatal conductance followed by R1, R2, and AT. However, in September, the results were skewed due to cloudy conditions. In the month of October, R2 showed a slightly higher stomatal conductance than R1, followed by the control and AT (*p* = 0.0334). In 2017, stomatal conductance differed significantly between months (Figure 3B; *p* = 0.0001) wherein, May showed a higher stomatal conductance followed by August and September, and lastly, June. There was no statistical difference between treatments (*p* = 0.174) or interaction between treatment and month (*p* = 0.8398).

#### 2.2.2. Leaf Temperature

**Figure 4:**
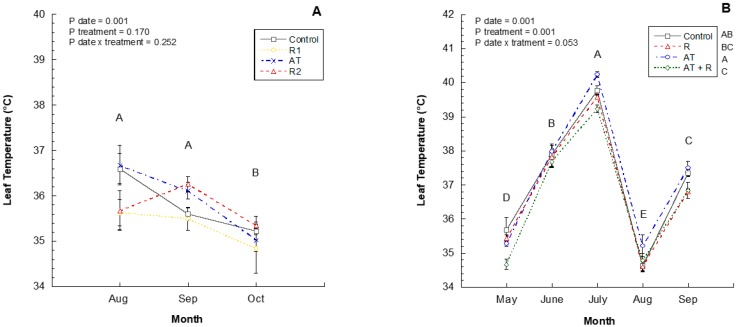
2016 and 2017 monthly leaf temperature (°C). (**A**) Leaf Temperature (°C) through the months of August to October 2016. 2016 Treatments: R1 = Reflectant at 9.35 L/ha; R2 = Reflectant at 18.7 L/ha; AT = Antitranspirant at 14 L/ha. (**B**) Leaf temperature from May to September 2017. 2017 Treatments: R = Reflectant at 9.35 L/ha; AT = Anti-transpirant at 9.35 L/ha; AT + R = Anti-transpirant at 9.35 L/ha and Reflectant at 18.7 L/ha. Bars represent ±1 standard error of the mean. Uppercase letters show significant differences between months.

Leaf temperatures were not impacted by treatments in 2016; however, there were significant differences between each month (Figure 4A). Leaves were cooler in October compared to September and August. Figure 4B shows that the 2017 leaf temperatures were significantly different each month (*p* = 0.0001), whereas in July, leaf temperatures were highest, followed by June, September, and May, and August had the lowest leaf temperatures. There was a significant effect of treatments on leaf temperature as well (*p* = 0.0001); where AT treated leaves had higher temperatures followed by Control, R and AT + R treatments respectively. There was no significant interaction between treatment and month (*p* = 0.0525).

#### 2.2.3. Relative Humidity

**Figure 5:**
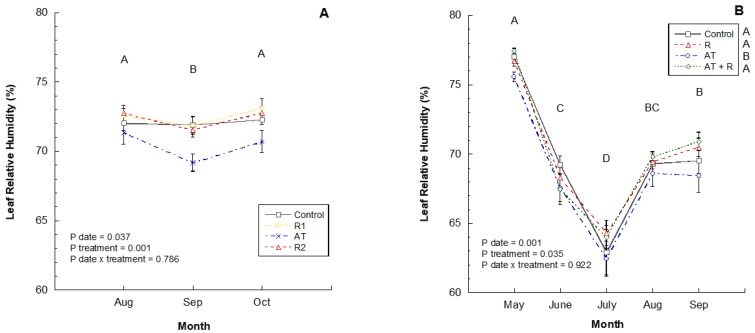
2016 and 2017 leaf relative humidity (%) by month. (**A**) Leaf relative humidity (%) through the months of August to October 2016. 2016 Treatments: R1 = Reflectant at 9.35 L/ha; R2 = Reflectant at 18.7 L/ha; AT = Anti-transpirant at 14 L/ha. (**B**) Leaf relative humidity (%) from May to September 2017. 2017 Treatments: R = Reflectant at 9.35 L/ha; AT = Antitranspirant at 9.35 L/ha; AT + R = Anti-transpirant at 9.35 L/ha and Reflectant at 18.7 L/ha. Bars represent ±1 standard error of the mean. Uppercase letters show significant differences between months.

In 2016 and 2017, leaf relative humidity readings were significantly different by month (*p* = 0.037, *p* = 0.0001, respectively). In 2016, August and October had higher humidity levels than September (Figure 5A) and in 2017, May had higher humidity, followed by September, August, June, and July (Figure 5B). In the 2016 study, there was no significant impact of treatments on relative humidity within the canopy. However, in 2017, treatments did impact the canopy relative humidity with AT treated trees having a lower humidity than all other treatments. 

### 2.3. Fruit Field Measurements

#### 2.3.1. Fruit Temperature by Treatment Orientation

In 2016 and 2017, the fruit temperature was significantly different between months (Table 1). The 2016 experiment showed that August had the highest temperatures and October the lowest temperatures. The treatments also had a significant impact on fruit temperature in 2016 (*p* = 0.0001), where R1 and AT treated fruit had the highest temperatures throughout the experiment, and the control and R2 had the lowest temperatures. In 2017, July had the highest fruit temperatures, followed by August and September. The treatments also had a significant effect on fruit temperatures (*p* = 0.0001), where the AT treated fruit had the highest temperatures throughout the 2017 experiment, followed by AT + R and the control, and R.

There was also a significant interaction effect of treatment on fruit temperatures during each month in the 2016 and 2017 experiments (Table 2). In August 2016, AT had the highest fruit temperatures followed by R1, R2, and the control. In September, R1 had the highest temperatures, followed by the control, AT, and R2. In October, R1 had higher temperatures followed by AT, R2, and the control. For 2017, Table 2 shows that the average fruit temperature was affected differently by treatment each month (*p* = 0.0336); in the month of July the Control, AT, and AT + R had the highest temperatures followed by R. In August, AT had the highest temperatures, followed by AT + R, the control and R had the lowest temperatures. In September, AT had the highest temperatures followed by the control, R, and AT + R.

Additionally, treatment had an impact on fruit temperatures based on the location of fruit on the tree. Fruit temperatures were affected by treatment on both the west and east sides of the canopy in 2016 (*p* treatment = 0.0055, 0.0003), respectively. However, there was only a treatment by date interaction on the west side of the canopy (*p* = 0.0046) compared to the east (*p* = 0.1332). When we analyzed fruit temperature by their orientation on the trees in 2017, there was a significant effect of month for fruit temperatures (*p* = 0.0001, 0.025, 0.0001, 0.0001; N, S, E, W, respectively). In July 2017, fruit on the east side had higher temperatures, followed by those on the S, N, and W sides of the trees. In August, the E orientation had the highest temperature, followed by N, S, and W. In September, S, E, and W had the highest temperatures, followed by N. There was no significant interaction of treatment and month in 2017 (*p* N, S, E, W = 0.1492, 0.1115, 0.439, and 0.1757, respectively). While treatment by date did not have a significant impact on any of the individual orientations, when fruit temperatures were averaged over the tree, there was a significant effect of treatment on fruit temperatures (Table 1; *p* = 0.0036). Here, the fruit temperatures were highest in the AT treatments in August 2017 and lowest in the control and R treatments, and the control, R, and AT + R treatments in September 2017.

#### 2.3.2. Sunburn Distribution and Severity

For 2016, average fruit sunburn on the outer canopy was significantly impacted by treatments (*p* = 0.0312) wherein AT and R2 had higher percentages of sunburn incidence followed by the control and R1. When separated by canopy orientation, the treatments showed a significant impact on the percentage of sunburn on the east side of the canopies (*p* = 0.0066), where AT and R2 showed a higher sunburn incidence, followed by the control and R1. The west side did not show any statistical significance between treatments for the percentage of sunburn (*p* = 0.2901; Table 2). For 2017, the percentage of fruit sunburn was significantly affected by month (*p* = 0.0001), where the months of August and September had the highest percentage of sunburn, and June and July had the least sunburn. Percentage sunburn was also significantly affected by treatment in 2017 (*p* = 0.0001), where the AT treated fruit had higher rates of sunburn than all of the other treatments. Treatments also influenced sunburn on the N and W sides of the tree canopy (*p* = 0.0121, 0.0001; N and W, respectively). However, percent sunburn was not significantly affected by treatment for each date for the averages or the location of fruit on the tree. It should be noted that due to field maintenance in 2017, some of the sunburn fruit dropped prior to the last sunburn assessment (Table 2). Overall, more sunburned fruit were found in treatments with the anti-transpirant in both 2016 and 2017. AT treatments increased sunburn by 6% in 2016 and 8% in 2017 when compared to their respective controls. In 2016, R1 treatments reduced sunburn by 10.9% when compared to the AT treatments and 4.9% when compared to the control. AT + R treatments reduced sunburn by 9.9% when compared to the AT treatment and 1.8% when compared to the control in 2017. 

### 2.4. Yield and Fruit Size Distribution

**Figure 6:**
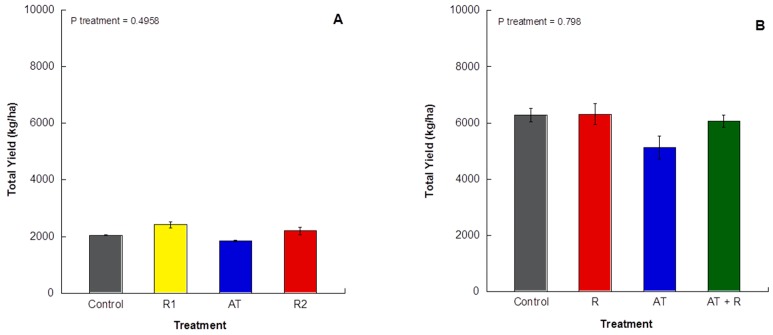
Total yield (kg/ha) of grapefruit harvest for 2016 and 2017. (**A**) 2016 total yield (kg/ha) from treated trees. (**B**) 2017 total yield (kg/ha) from treated trees. Bars represent ±1 standard error of the mean.

For 2016 and 2017, there was no significant impact of treatment on total yield (Figure 6A; *p* = 0.4958).

For 2016, Figure 7A shows that treatments significantly affected fruit size, (*p* = 0.0001). AT and the control trees had the highest amount of smaller sized fruit when compared to the R1 and R2 (categories 112 and 96). The R1 and R2 treatments had more fruit in the larger size categories (categories 64 and >64). For 2017, Figure 7B shows that fruit size distribution was not significantly affected by treatments (*p* treatment = 0.65). 

## 3. Discussion

Physiological disorders are often caused by the response of fruit tissue to biotic and abiotic stresses that include environmental conditions, temperature, relative humidity, plant water status, nutritional status, chemicals, etc. To manage these factors and to be able to prevent physiological disorders, it is important to understand how the environment interacts with the plant to cause the response and resulting disorder. Disorders on the surface of citrus fruits are associated with the rupture of glands, phytotoxic injury to tissues, and subsequent water loss [28]. Weather related causes of fruit damage include frost, wind, rind disorders, and sunburn. Sunburn damage occurs when fruit surface temperatures and light intensities are very high and the peel develops burn injury in the location facing high solar radiation. Sunburn can have immediate and secondhand impacts on fruit physiology and fruit quality. Managing sunburn requires system approaches that combine good orchard management and effective sunburn management strategies. Strategies for sunburn control include increasing evaporative cooling, protective netting, protectant sprays such as kaolin-based particle films, and calcium carbonate particle films [9,13,25,29,30,31]. Since excess light stress is often associated with water stress and high temperatures, these factors can also influence the incidence of sunburn. High temperatures combined with low RH are frequently associated with stomatal closure [32] reduced leaf transpiration and photosynthesis. While we did not observe treatment effects on canopy temperatures, effects at the fruit or leaf level may be more important to photosynthetic functioning and transpiration. 

### 3.1. Tree Measurements

Although trees were not water stressed because of adequate irrigation, they showed symptoms of sunburn in all treatments as a consequence of high temperature, solar radiation, and low relative humidity recorded in the summer months. Sunburn was significantly related to lower stomatal conductance and relative humidity. It is well established that high solar radiation and high temperature lead to lower stomatal conductance [11,19,22,29]. These conditions are more prevalent during the summer months in south Texas, which explains the observed sunburn despite a lack of water stress.

In the 2016 and 2017 experiment, stomatal conductance followed inverse patterns to that of temperatures; as the temperatures increased, stomatal conductance decreased, but higher relative humidity was associated with higher stomatal conductance. This indicates that trees closed their stomata to conserve water in times of higher temperatures [33]. Del Amor et al. (2010) [34] mention that anti-transpirants can regulate transpiration, but limit CO_2_ exchange, which leads to the reduction of stomatal conductance, but found that under water stressed conditions, stomatal conductance, transpiration, and photosynthesis were reduced. Reflectants like kaolin have been shown to reduce the canopy temperatures through the reflectance of infrared and ultraviolet radiation as well as PAR [35]. Kaolin sprays have also been shown to increase stomatal conductance in grapefruit leaves, but reduced photosynthesis due to photoinhibition [36]. In these studies, the reflectant treatments did show some influence in increasing stomatal conductance when compared to the AT treatments, but only in 2016. As we did not see a significant change in canopy temperatures, it may indicate that the sprays were not frequent enough or that the rates were not high enough to cause a reduction in the overall canopy temperatures. In addition to this, the weather and environmental conditions in 2016 were significantly different than those experienced in 2017. In 2016, the summer months were cloudy and there were more frequent rainstorms compared to the subsequent year.

Leaf temperatures can be affected by transpiration rates, with higher temperatures increasing the rate of transpiration to a certain point [37]. This increase in transpiration may have evaporative cooling effects on trees [38]. If transpiration decreases due to stomatal closure, plant temperature can increase, but the plant conserves water [38,39,40]. The moisture content of the air can also alter the temperature of the plants through cooling of the leaves. The effect of humidity upon leaf temperatures is greater at higher air temperatures because vapor pressure is greater and stomata are more open, so that more transpirational cooling occurs [41]. In these experiments, leaf temperature and relative humidity seemed to be inversely proportional; when temperatures increased, the relative humidity and stomatal conductance decreased. Relative humidity is dependent on temperature, and higher temperatures generally lead to increased transpiration. Kaolin and calcium treatments have been used to reduce plant leaf temperatures and reduce stress in crops [35]. In experiments conducted by Glenn et al. (2002) [42], they found that kaolin treatments reduced leaf and fruit temperatures of Fuji apple by 8 °C. In 2017, the increased temperatures in the AT application indicate that decreases in evaporative cooling through the stomata could have possibly been caused by this treatment, which is supported by Nammah (1979) [43], who found that AT treatments influenced leaf temperatures. While AT treatments reduced stomatal conductance and potentially conserved water, they had a negative influence on leaf temperature, which could eventually affect the plant. Alternatively, reflectants reduced leaf temperatures in 2017, most significantly in the AT + R treatments, indicating that the temperature effects of the AT can be mitigated with strategic applications of reflectants. 

### 3.2. Fruit Physical Effects

Ambient temperatures that reach 30–36 °C and increasing fruit temperatures of 38–48 °C can lead to sunburn fruit in citrus [4]. These experiments were performed in trees with north to south facing rows, where temperature distribution varied throughout the canopy. The temperatures of the leaves and fruit were highest on the west side of the tree canopies and this, in turn, influenced the higher incidence of sunburn. Row orientation can influence fruit leaf and canopy temperatures and light interception [44]. The decrease in temperature and sunscald linked to row orientation facing north and south is thought to be due to shading from neighboring plants in the same row during critical sun exposure [1]. This can then affect the distribution of sunburn in the outer canopies. As temperatures increased throughout the year, there was a corresponding increase in sunburn, which then tapered off in September. As the highest fruit, leaf, and ambient temperatures were found during the July and August months, the sunburn incidence corresponded directly to these findings. 

Treatments did affect sunburn incidence and the temperatures of fruit, however, the threshold for sunburn has been shown to be higher in other fruit [9,13,45]. However, we did not have continuous monitors available for fruit and leaf temperature, so there is a likely chance that the temperatures exceeded what we recorded at midday. Treatments affected both fruit temperatures and sunburn incidence, with more sunburn found in fruit treated with the anti-transpirant. Furthermore, AT treated fruit showed a higher incidence of leaf and fruit temperatures throughout the studies, indicating that this treatment should not be applied during times of high temperatures and solar radiation unless it is paired with a reflectant to mitigate some of the negative effects. In contrast to our results, a study by Abdallah (2019) [46] showed that AT treatments had no significant effect on the sunburn of tomato. This is similar to the results found by Tsai et al. (2013) [10], who found low rates of sunscald in treatments containing calcium carbonate (CaCO_3_) as well as low leaf temperatures, and a low net photosynthetic rate due to particles inhibiting gas exchange. 

### 3.3. Yield

Yield was not significantly affected by any of the treatments in both years, however, numerically, the yields were lower in trees treated with the AT. In 2017, the AT + R treatments seemed to mitigate the negative impact of the AT, increasing yield and fruit mass to the levels of other treatments. Contrary to these findings, Abdallah (2019) [46] found that AT treatments with pinolene had lower yields compared to those with kaolin particle films in tomato. Brillante et al. (2016) [30] also compared kaolin particle films with film forming anti-transpirants in grapes and found that while the kaolin treatments increased yields and water use efficiency, AT decreased sugars and fruit quality. These differences could be attributed to the high water content of tomato and grapefruit, which often determine fruit weight and size related yield.

## 4. Materials and Methods 

### 4.1. Experimental Setup and Site Information

**Figure 8:**
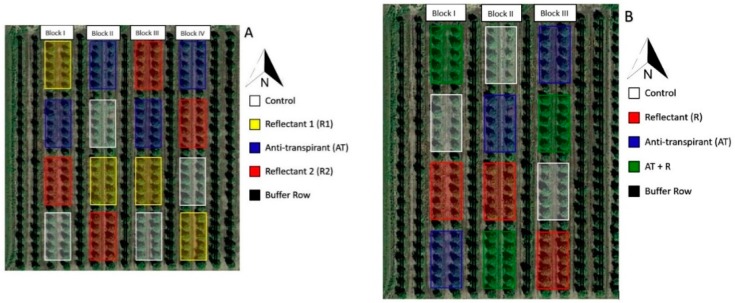
Experimental block setup for the sunburn mitigation trials. (**A**) 2016 experimental setup. (**B**) 2017 experimental setup.

These studies were conducted in the 2016 and 2017 growing seasons in a 7-year-old grapefruit (*Citrus paradisi cv.* Rio Red) grove located at the Texas A&M University-Kingsville Citrus Center in Weslaco, Texas (Hidalgo County, 26.131893, −97.948739). This region is considered semi-arid and is characterized by a subtropical climate. Soil types in this grove include Mercedes clay, Hidalgo sandy clay loam, and the Raymondville clay loam series, with the majority composed of Mercedes clay (~50% clay particles). Tree orientation was north to south and the planting density was 299 trees per ha. Each year had a different experimental layout and different treatment application rates (Figure 8). In 2016, different rates of reflectant and anti-transpirant were compared to the control. In 2017, one rate was used for each chemical, but different combinations of the reflectants and anti-transpirant were evaluated to determine if better efficacy could be achieved. 

The 2016 experimental plot was divided into four blocks that consisted of two rows of trees per block. Each block contained 40 trees with a row of buffer trees between each block. Four treatments were applied within each block, and each treatment was applied to 10 trees in two separate, but adjacent rows (five trees per row) for a total of 40 trees per treatment as shown in Figure 8A. The 2017 plot was divided into three blocks that consisted of two rows of trees per block with a north–south row orientation. Each block contained 40 trees, with a row of buffer trees between each row. Four treatments were applied within each block, and each treatment was applied to 10 trees in two separate, but adjacent rows (five trees per row) for a total of 30 trees per treatment as shown in Figure 8B. After the initial trial (2016), the number of blocks was reduced in the subsequent trial to allow for time limitations due to the necessity of collecting consistent samples within a restricted time frame. The randomization was changed in the second year to reflect a different combination of the treatment chemicals. 

### 4.2. Treatments

In 2016, two rates of a calcium-based reflectant (Reflectant 1 and Reflectant 2), an anti-transpirant (AT), and a water control were applied to the trees (Table 3). The reflectant was a proprietary blend composed of 30% calcium acetate and carbonate in a suspension formulation. The anti-transpirant composition was a proprietary blend of 96% di-1-*p*-methene with 4% pinolene. In 2017, the same products were used, but in different combinations: anti-transpirant (AT), reflectant (R), anti-transpirant and reflectant (AT + R), and a control (Table 3). In 2016, treatments were applied only once (August), whereas in 2017, three applications were made (May, June, and July) using the rates shown in Table 3.

### 4.3. Field Measurements

Field measurements were taken regularly before the first application and after each subsequent treatment throughout the two growing seasons. In 2016, stomatal conductance, fruit and leaf temperature, and sunburn assessment on the east and west sides of the trees were measured. A week before fruit harvest (10/28/2016), 10 fruits were collected from three trees per treatment by block for a total of 120 grapefruit from each treatment were randomly selected for destructive and nondestructive measurements. Similarly, in 2017, stomatal conductance, fruit and leaf temperature, sunburn assessment on all four sides of the tree, fruit set, and fruit drop were measured. Field measurements were taken monthly throughout the summer growing season until harvest. 

In 2016, stomatal conductance was recorded monthly with a leaf porometer (Decagon Devices, Pullman, WA, USA) from August to October, and in 2017, stomatal conductance was recorded monthly from May to September. A random, fully expanded, recently mature leaf from each tree was selected for measurement. The porometer was placed on the center of the leaf blade, avoiding the midrib, and repeated for six trees per treatment in each block for a total of 24 trees per treatment in 2016 and 18 trees per treatment in 2017. 

Additionally, leaves were randomly selected, collected, washed, and processed to measure leaf area with an LI-3100 area meter (LI-COR Industries, Lincoln, NE, USA) for the months of May, June, July, and September. 

Fruit and leaf temperatures were recorded with a digital thermometer and thermocouple on the east (E) and west (W) side of each tree during peak temperatures and at the Sun’s zenith (approximately midday) in 2016. One grapefruit from every tree at the two cardinal directions was measured for a total of 40 trees per treatment and four fruits per tree. In 2017, fruit temperature was recorded on the N, S, E, and W side of each tree and one grapefruit per orientation was measured for a total of 30 trees per treatment and four fruits per tree.

Ambient air temperatures and humidity were recorded continuously at hourly intervals using portable sensor datalogger modules (Model: HOBO waterproof shuttle, Onset, Cape Cod, MA, USA) placed within the canopy of two trees per treatment per block. 

### 4.4. Fruit Field Measurements

Fruit sunburn incidence (number of fruits affected) was recorded on the outer canopy of each tree using a 0.26 m² (2.85 ft²) polyvinyl chloride (PVC) grid on randomized locations within each E and W quadrant of each tree in 2016 and the N, S, E, and W quadrants in 2017. Ten trees per treatment in each block were monitored for sunburn each month throughout the experiment. In addition, a complete sunburn fruit count by quadrant was conducted in October 2017. 

### 4.5. Fruit Harvest and Yield

Fruit were harvested each year from six trees per treatment by hand harvesting all fruits and placing in separate bins for subsequent measurements. Bins were weighed and the fruit were sized using a sizing machine (Wilde Manufacturing Co., Bailey, MI, USA). The number of fruits in each size class as well as fresh mass were recorded. Prior to harvest, a representative subsample was collected from each treatment and block, and used to determine fruit weight, rind thickness, brix, acidity, and maturity index. 

## 5. Conclusions

While trees were well irrigated and did not show significant water stress in general, high leaf temperatures, solar radiation, and low relative humidity observed in summer months can lead to the development of sunburn, regardless of plant water status. As leaf temperatures increased, leaf relative humidity and stomatal conductance declined, even though trees were well irrigated. Overall, leaf temperatures were highest in anti-transpirant treatments, and leaf relative humidity was lowest in these treatments. Reflectants sprayed after the anti-transpirant treatments counteracted the effects of the anti-transpirants, lowering leaf temperatures and increasing relative humidity. More sunburned fruit were found in treatments with the anti-transpirant in both 2016 and 2017. Reflectant treatments reduced sunburn incidence by 4.9% compared to the control in 2016 and 1.8% in 2017. Compared to the anti-transpirant treatment, the sunburn incidence was lowered with reflectant treatments by 10.9% and 8% for 2016 and 2017, respectively. 

As expected, more sunburn occurred during times of high fruit and leaf temperatures. However, reflectant application reduced sunburn by reducing fruit temperatures and blocking light from the fruit surface. While anti-transpirants alone can increase sunburn and leaf temperatures, these negative effects can be mitigated by the application of a reflectant.

## Figures and Tables

**Figure 7:**
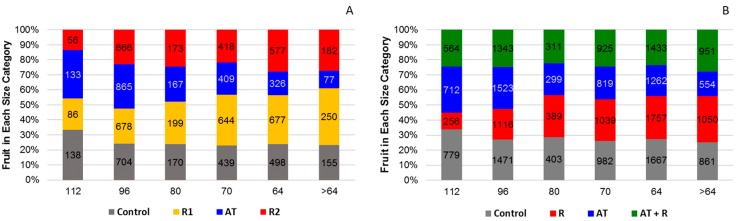
Percentage of fruit in each size category by treatment for 2016 and 2017. (**A**) Percent of fruit in each size category for 2016. (**B**) Percentage of fruit in each size category for 2017.

**Table 1:** Fruit temperature (°C) month for 2016 and 2017. Lowercase letters represent significant differences between treatments for each month.

		Fruit Temperature (°C)				
		Tree Orientation				
Month-Year	Treatment	N	W	S	E	Average
**August-16**	Control		31.67 b		29.41	29.92 de
	R1		34.34 a		33.29	33.71 ab
	AT		34.87 a		32.94	33.91 a
	R2		32.24 b		31.35	31.73 c
**September-16**	Control		28.85 c		34.00	31.53 cd
	R1		29.07 c		34.57	32.05 bc
	AT		27.57 cd		34.03	30.80 cde
	R2		27.54 cd		32.49	29.93 de
**October-16**	Control		25.58 e		30.61	28.11 f
	R1		26.79 de		33.95	30.30 cde
	AT		26.23 de		33.43	29.83 def
	R2		26.48 de		32.70	29.61 ef
*p* date =			0.0001		0.0025	0.0001
*p* treatment =			0.0055		0.0003	0.0001
*p* date x treatment =			0.0046		0.1332	0.0011
**July-17**	Control	37.54	37.19	37.78	37.98	37.62 ab
	R	36.77	36.61	37.35	37.65	37.10 bc
	AT	37.57	37.37	37.95	38.31	37.80 ab
	AT + R	37.07	37.42	37.44	37.57	37.37 ab
**August-17**	Control	35.78	35.05	36.27	36.82	35.98 d
	R	35.29	35.10	36.08	37.33	35.95 d
	AT	37.54	37.25	37.98	38.53	37.82 a
	AT + R	35.80	35.45	37.30	37.64	36.55 cd
**September-17**	Control	35.11	36.03	36.68	36.01	35.96 d
	R	34.60	35.64	37.20	36.10	35.89 d
	AT	35.48	36.73	37.02	36.45	36.42 cd
	AT + R	34.86	36.25	37.43	36.16	36.18 d
*p* date =		0.0001	0.0001	0.0250	0.0001	0.0001
*p* treatment =		0.0001	0.0004	0.0413	0.013	0.0001
*p* date x treatment =		0.1492	0.1757	0.1115	0.4396	0.0336

**Table 2:** Percent fruit sunburn in 2016 and 2017. Lowercase letters represent significant differences between treatment and date for each column.

		Fruit Sunburn				
		Tree Orientation				
Month-Year	Treatment	N	W	S	E	Average
**October-16**	Control	-	22.15	-	12.53 b	17.34 ab
	R1	-	14.25	-	10.63 b	12.44 b
	AT	-	22.97	-	23.79 a	23.38 a
	R2	-	18.04	-	24.40 a	21.22 a
	*p* treatment =	-	0.2901	-	*0.0066*	*0.0312*
**June-17**	Control	0	0.893	1.333	0	0.566
	R	0	0	1.786	0	0.455
	AT	0	8.588	2.407	0.714	3.032
	AT + R	0	7.25	2.906	0	2.584
**July-17**	Control	0	2.414	0	0	0.631
	R	0	0	0	0	0
	AT	1.149	6.121	2.381	0	2.456
	AT + R	0	2.778	0	0	0.718
**August-17**	Control	0	14.236	4.138	11.852	7.481
	R	0	13.678	7.56	11.548	8.173
	AT	2.989	29.32	12.644	18.21	15.628
	AT + R	0	10.611	7.373	4.833	5.704
**September-17**	Control	1.149	12.245	5.172	5.714	6.073
	R	0	15.536	13.393	5.172	8.496
	AT	1.235	27.637	12.069	8.046	12.168
	AT + R	0	13.448	7.685	4.425	6.335
*p* date =		0.4191	*0.0001*	*0.0001*	*0.0001*	*0.0001*
*p* treatment =		*0.0121*	*0.0001*	0.094	0.2533	*0.0001*
*p* date x treatment =		0.3866	0.2212	0.7647	0.5043	*0.1083*

**Table 3:** Product sprays applied at various dates and concentrations in the 2016 and 2017 growing seasons *.

**2016 Treatment**	**Application Rate**	**May**	**June**	**July**	**Aug.**
Reflectant 1 (R1)	9.35 L/ha/265 L water	-	-	-	X
Anti-transpirant (AT)	14 L/ha/378 L water	-	-	-	X
Reflectant 2 (R2)	18.70 L/ha/265 L water	-	-	-	X
Control	Water	-	-	-	X
**2017 Treatment**	**Application Rate**	**May**	**June**	**July**	**Aug.**
Anti-Transpirant (AT)	9.35 L/ha/378.5 L water	X	X	X	-
Reflectant (R)	18.70 L/ha/265 L water	X	X	X	-
Anti-transpirant	9.35 L/ha/378.5 L water	X	X		-
Reflectant (AT + R)	18.70 L/ha/265 L water	-	-	X	-
Control	Water	X	X	X	-

* X on table indicates month of treatment.
